# Acquired Angioedema and Chronic Lymphocytic Leukemia: Unraveling the Complex Interplay and Addressing Refractory Cases

**DOI:** 10.7759/cureus.50238

**Published:** 2023-12-09

**Authors:** Divya Shah, Radha Rishi

**Affiliations:** 1 Internal Medicine, University of Arizona College of Medicine-Phoenix, Phoenix, USA; 2 Allergy and Immunology, Arizona Allergy Associates, Phoenix, USA

**Keywords:** angioedema management, c1 esterase inhibitor deficiency, c1 esterase inhibitor, chronic lymphocytic leukemia (cll), acquired angioedema

## Abstract

Acquired angioedema (AAE) due to deficiency of a C1 esterase inhibitor (C1-INH; AAE-C1-INH) is a rare and potentially fatal syndrome characterized by recurrent episodes of angioedema without urticaria. Often underdiagnosed due to its rarity and mimicry of common allergic reactions, AAE-C1-INH is associated with lymphoproliferative disorders, necessitating early recognition for improved outcomes. We present a case of a 63-year-old male diagnosed with AAE-C1-INH and concurrent stage 0 chronic lymphocytic leukemia (CLL), a rarely documented association. Despite chemotherapy, the patient experienced persistent angioedema until C1 esterase inhibitor therapy was initiated. This case underscores the importance of screening for lymphoproliferative disorders in AAE-C1-INH patients and explores refractory cases, urging further research into mechanisms and treatment strategies.

## Introduction

Angioedema, characterized by vasodilation and increased vascular permeability, can be hereditary or acquired. Hereditary angioedema is an autosomal dominant disorder defined by a functional C1 esterase inhibitor protein deficiency. A deficiency in C1-INH characterizes acquired angioedema (AAE) via consumption or inactivation, increased activation of the classical complement pathway, and bradykinin-mediated edema [1]. Increased catabolism in AAE-C1-INH may be related to an autoimmune condition (e.g., systemic lupus erythematous) or a lymphoproliferative process (e.g., monoclonal gammopathy of unknown significance, chronic lymphocytic leukemia, multiple myeloma, non-Hodgkin lymphoma) [1].

Chronic lymphocytic leukemia, a B-cell lymphoproliferative disorder, is characterized by the progressive accumulation of functionally incompetent lymphocytes, and its diagnosis may pose challenges due to variable and often benign clinical presentations [2]. The link between CLL and AAE highlights the importance of recognizing this association, as AAE may be associated with CLL, necessitating comprehensive evaluation and management strategies [3]. This article presents a case highlighting the necessity of CLL screening in AAE-C1-INH cases and discusses the rarity of refractory AAE-C1-INH following CLL treatment. This case was previously presented as a poster at the 2023 American College of Allergy, Asthma, & Immunology Annual Meeting on November 10, 2023, and then published as a brief abstract in the supplement of the November 2023 edition of the Journal of Allergy and Clinical Immunology.

## Case presentation

We present a 63-year-old male with a history of allergic rhinitis who experienced recurrent episodes of lip and foot swelling over the past decade, initially attributing them to allergic reactions. As the frequency of swelling episodes increased, occurring up to three times a month, the patient sought medical attention for asymmetric upper and lower lip swelling. Typically, these episodes spontaneously resolve within a few days. Before the onset of symptoms, he had no personal or familial history of angioedema during childhood or adolescence and no history of food allergies or new medications. He denied experiencing fever, rashes, weight loss, joint pain, or difficulty swallowing or breathing. Furthermore, he had never used angiotensin-converting enzyme inhibitors or angiotensin receptor blockers, had no history of smoking or alcohol use, and had not been admitted to a hospital within the last decade.

**Figure 1:**
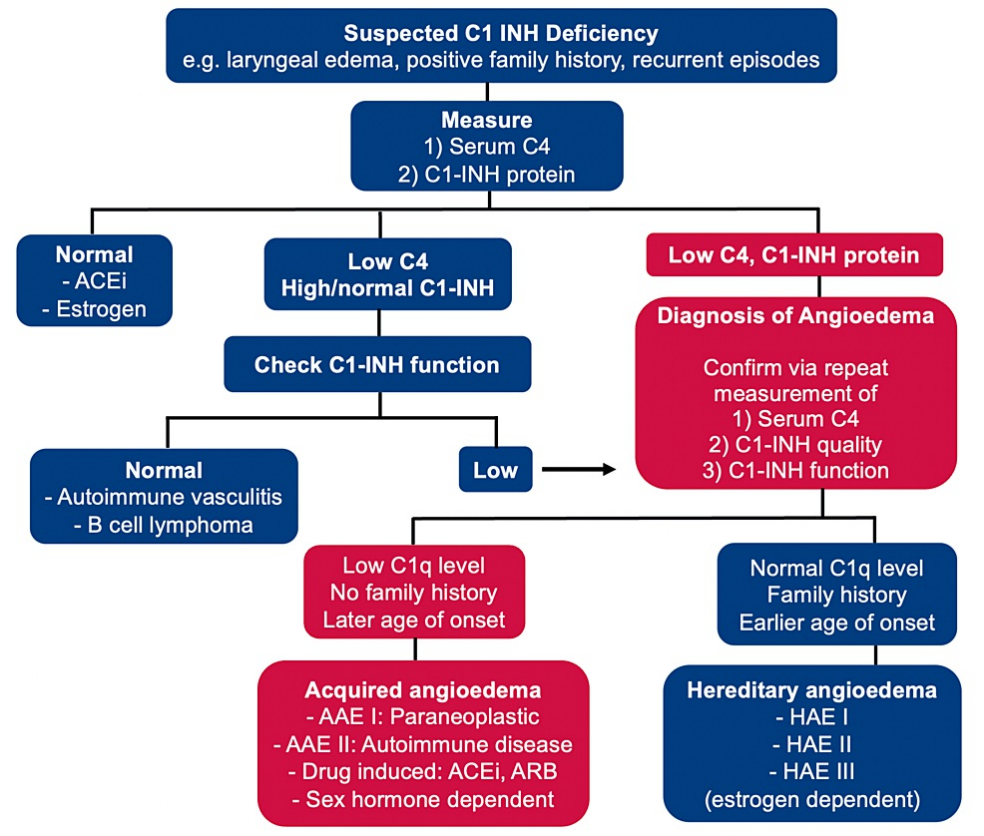
Diagnostic approach to C1-INH deficiency ACEI: angiotensin-converting enzyme (ACE) inhibitors, ARB: angiotensin receptor blockers

The patient’s vital signs were within the normal range during the clinic visit. The physical examination showed clear lungs without stridors and asymmetric upper and lower lip swelling without hives. All other systems examined were normal. Routine allergy testing revealed an allergy to dog dander, but the patient reported no recent pet exposure. Laboratory results were unremarkable, including a complete blood count, electrolyte panel, and rheumatologic antibody screening. However, further investigation indicated low levels of C3, C4, and C1 esterase inhibitors and reduced C1 esterase inhibitor function (Table 1). With negative C1-INH autoantibodies, the patient was diagnosed with acquired angioedema due to C1 esterase inhibitor deficiency (Figure 1). Methyltestosterone, a synthetic anabolic steroid derived from testosterone, was prescribed to manage symptoms by increasing C1-INH and C4 serum levels, a strategy commonly used in hereditary angioedema, with no improvement [4].

**Table 1:** Key laboratory values prior to initiating chemotherapy mg: miligrams; dL: deciliter; IU: international units; U: units

Lab name	Lab value	Reference range
C3 complement component	57 mg/dL	90-180 mg/dL
C4 complement component	<2 mg/dL	16-47 mg/dL
C1 esterase inhibitor protein	8 mg/dL	21-39 mg/dL
Immunoglobulin E	104 IU/mL	<100 IU/mL
C1 esterase inhibitor function	59%	>68%
C1Q complement component	<3.6	5-8.6 mg/dL

**Figure 2:**
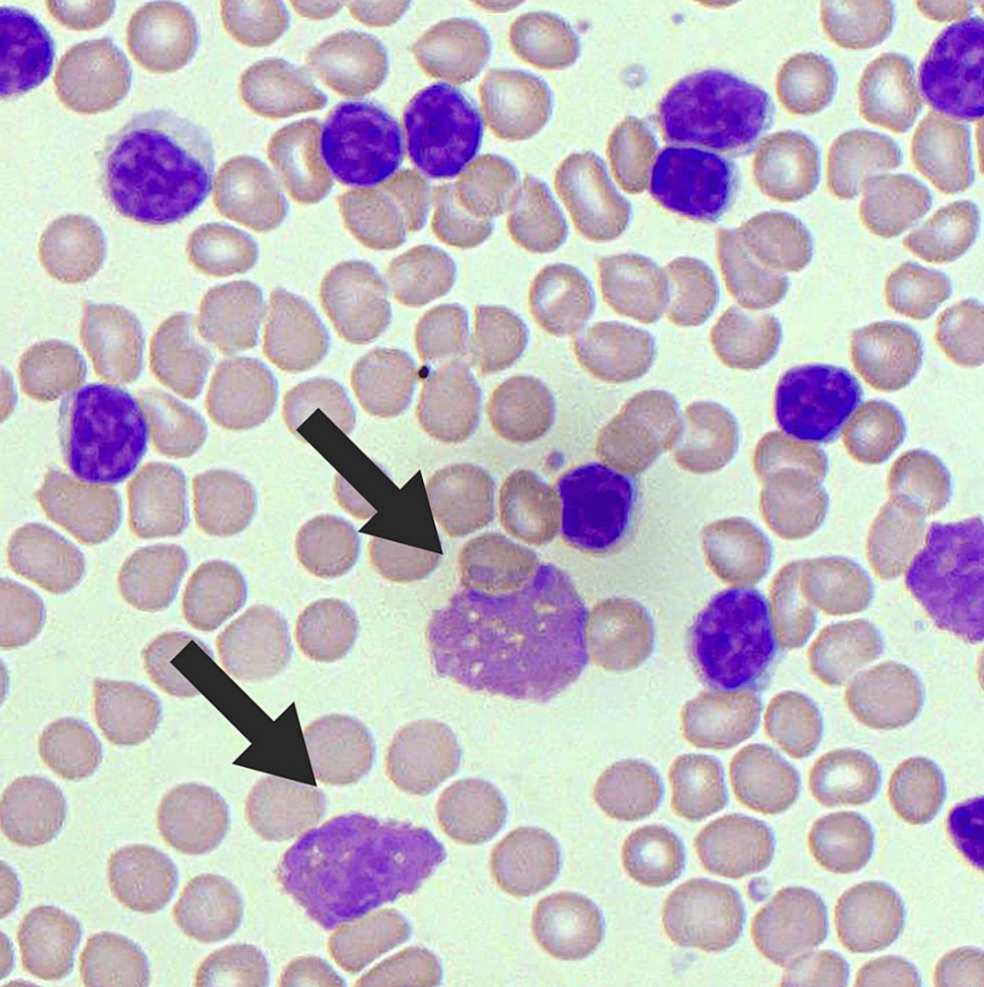
Smudge cells are characteristic of chronic lymphocytic leukemia

Following the initial diagnosis of AAE-C1-INH, the patient underwent additional laboratory testing to determine the underlying cause. Initial infectious and endocrinology labs were all negative or within the normal range. With an unidentified cause for AAE-C1-INH, additional investigation, including a bone marrow biopsy, revealed multiple smudge cells, confirming stage 0 chronic lymphocytic leukemia (Figure 2) [5]. Although the complete blood count showed no significant abnormalities, suggesting early-stage CLL, the occurrence of angioedema led to the initiation of rituximab treatment for the underlying CLL. After initiating chemotherapy, the patient exhibited no evidence of CLL relapse on routine testing and imaging. Post-chemotherapy, the patient’s C1 esterase inhibitor (C1-INH) levels increased to 16 mg/dL, with a C1-INH function of 65%. Despite the improvement, these levels did not normalize, and the patient reported reduced but persistent angioedema episodes. To address this, the patient commenced C1 esterase inhibitor therapy, typically approved for hereditary angioedema, at 1000 units three times per week under compassionate use approval. This treatment has effectively controlled the patient’s symptoms for the past decade.

## Discussion

This article emphasizes that when diagnosing acquired angioedema, clinicians should conduct a comprehensive workup to explore various potential causes, including paraneoplastic processes, autoimmune conditions, medication reviews, and other relevant factors. This article specifically underscores the established association between acquired angioedema due to C1 esterase inhibitor deficiency and lymphoproliferative disorders, emphasizing the importance of screening for neoplastic disease, such as B-cell disorders, in the clinical workup of AAE (Figure 1). The mechanism by which lymphocytic cells induce C1-INH deficiency, triggering angioedema, is not fully understood, with the prevailing theory implicating anti-C1-INH autoantibodies [6]. Despite the common occurrence of these autoantibodies in AAE-C1-INH patients, the presented case exhibited undetectable levels. The article advocates for further research into the mechanisms of AAE-C1-INH induced by lymphoproliferative disorders. Studies, including one by Castelli et al., reveal a significant correlation between AAE-C1-INH and lymphoproliferative disorders, emphasizing the complexity of this association [3]. The suggested alteration in B-cell proliferation control may contribute to the development of AAE. Per Figure 1, paraneoplastic processes, autoimmune conditions, medications, etc., may play a role in the development of AAE. This case emphasizes the need for a thorough, systematic clinical evaluation of patients with AAE, as their presentation may be secondary to neoplastic disease. The article recommends routine screening for neoplastic processes, such as lymphoproliferative disorders, in AAE-C1-INH cases, with a particular emphasis on considering a bone marrow biopsy for cases with an unknown etiology. Awareness of this rare condition and anticipation of connections, such as between chronic lymphocytic leukemia and AAE, can facilitate early diagnosis and treatment of the underlying disease, leading to more favorable patient outcomes.

The article discusses a rare case of acquired angioedema due to C1 esterase inhibitor deficiency refractory to medical management of the underlying condition, specifically CLL. Despite chemotherapy, the patient experienced persistent angioedema, challenging the belief that successful treatment of the underlying cause should alleviate angioedema episodes. The article suggests that chemotherapy may not have induced a sufficient immunosuppressive state to eliminate angioedema episodes independently of its impact on CLL [7]. Notably, the lack of normalization in C1-INH levels and function after chemotherapy supports this theory. The article proposes further investigation into the immune system aspects contributing to recurrent angioedema flares. The use of C1 esterase inhibitory therapy, extrapolated from hereditary angioedema treatments, proved effective in managing persistent angioedema in this case [8]. However, the absence of approved therapies for AAE-C1-INH poses a challenge, as patients may resist available treatments. The article underscores the need for additional research to uncover the mechanism behind refractory angioedema in AAE patients and to develop targeted medications for AAE-C1-INH.

## Conclusions

The article highlights the significant morbidity and mortality associated with acquired angioedema due to C1 esterase inhibitor deficiency (AAE-C1-INH). It provides another instance supporting the connection between AAE-C1-INH and lymphoproliferative disorders, underscoring the importance for clinicians to include lymphoproliferative disorders in their differential diagnoses when encountering unexplained angioedema cases. The presented case represents one of the initial reported instances of acquired angioedema refractory to treatment of the underlying disorder. This emphasizes the need for further research to ascertain whether this challenging scenario is observed in other cases, emphasizing the complexity of AAE-C1-INH and the necessity for a thorough understanding and exploration of potential treatment approaches.
